# Lumbar hernia Jean-Louis Petit: A case report and literature review

**DOI:** 10.1016/j.radcr.2025.05.068

**Published:** 2025-06-26

**Authors:** Asmae Guennouni, Aya Laridi, Chaimae Abourak, Soukaina Bahha, Nabil Moatassim Billah, Itimas Nassar, Kaoutar Imrani

**Affiliations:** Central Radiology Department, Ibn Sina University Hospital, Morocco

**Keywords:** Jean Louis Petit, Lumbar hernia, Ultrasound, CT

## Abstract

Inferior lumbar hernias are exceptionally rare, representing a small fraction of all abdominal wall hernias. Among them, hernias of Jean-Louis Petit, occurring through the inferior lumbar triangle, are particularly uncommon and often overlooked in clinical practice. We present the case of a 53-year-old woman with no significant medical history who developed a painless, progressively enlarging mass in the right lumbar region. Ultrasound revealed a hernial sac containing omental and digestive structures, which was further characterized by CT imaging as an inferior lumbar hernia containing epiploic fat and bowel loops, with no evidence of strangulation. The patient was referred for elective surgical repair to prevent future complications. This case underscores the importance of recognizing lumbar hernias as a differential in atypical flank masses and illustrates the critical role of CT in accurate diagnosis and surgical planning. Given the rarity of this entity, increased awareness among clinicians and radiologists is essential for timely and appropriate management.

## Introduction

Lumbar hernias account for less than 2% of all hernias, with only 5% being inferior lumbar hernias. This makes hernias of Jean-Louis Petit one of the rarer forms of hernias [1].

Small hernias are asymptomatic, whereas larger ones present as tender masses causing lower back pain. The diagnosis is confirmed through CT scans [2]. Early and accurate diagnosis is crucial, as delayed management increases the risk of complications. Imaging modalities play a pivotal role in both identifying the hernia and planning surgical intervention.

## Case report

**Fig. 1:**
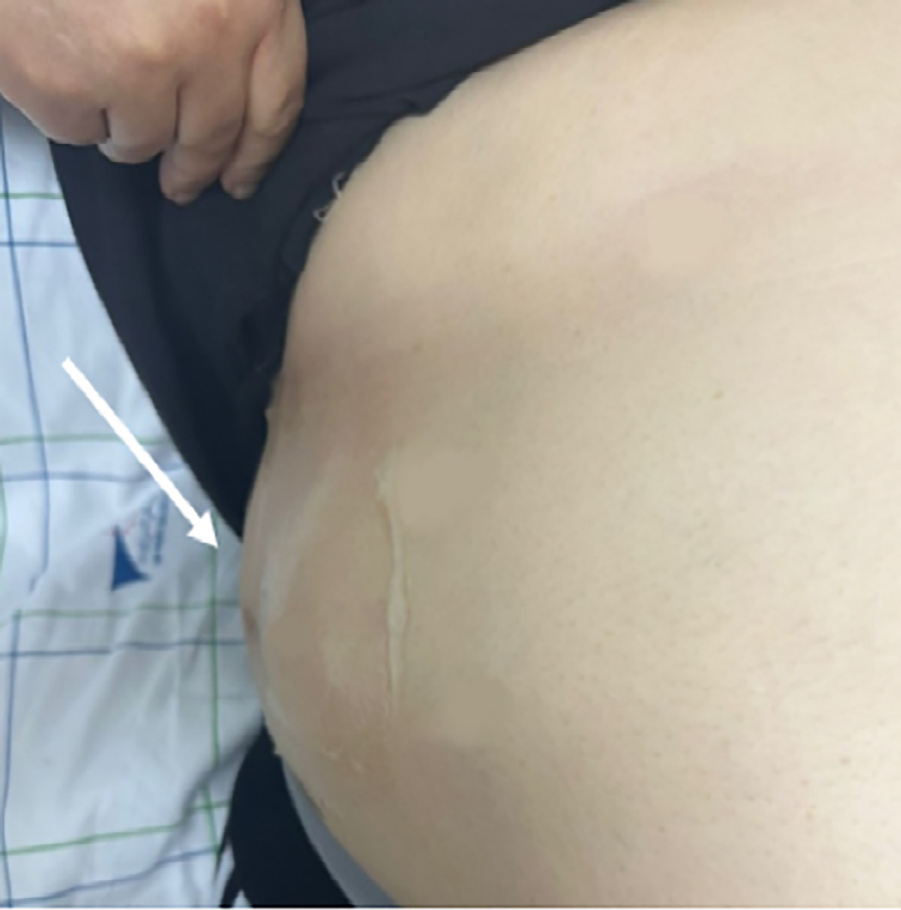
Clinical imaging of the patient shows a compressive and reducible swelling in the lumbar region (white arrow).

We report the case of a 53-year-old woman with no significant medical history who presented with a gradually enlarging right lumbar mass (Fig. 1). The mass had been present for several months and was painless, with no associated symptoms such as fever, weight loss, gastrointestinal disturbances, or urinary changes.

On physical examination, a soft, reducible swelling was noted in the right lumbar region. The mass increased in size when the patient was standing or performing the Valsalva maneuver and reduced when she was in a supine position. There were no signs of inflammation, erythema, or overlying skin changes. No tenderness was elicited upon palpation, and no evidence of bowel obstruction was found.

**Fig. 2:**
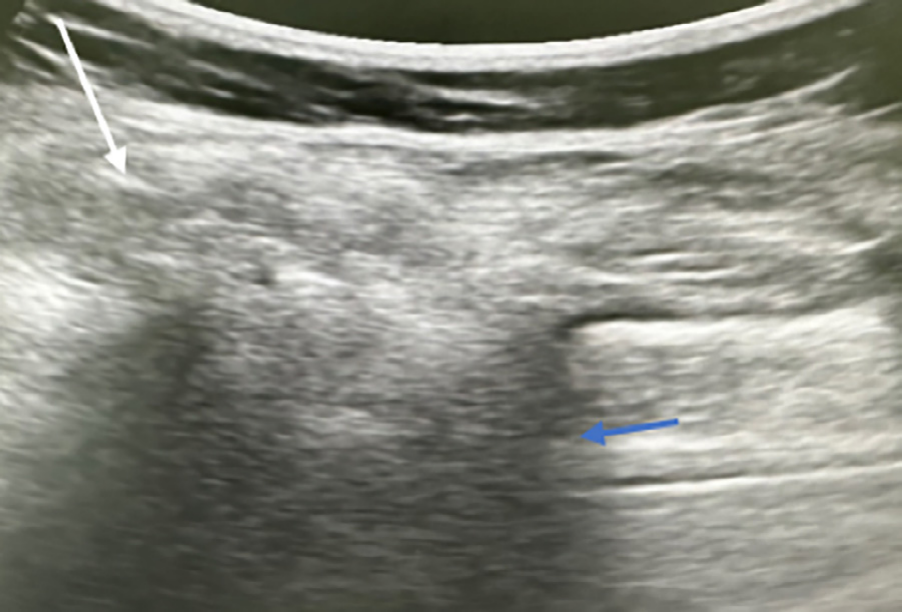
Ultrasound imaging shows a hernial sac containing omental and digestive structures (white arrow) with a wide neck (blue arrow).

An initial ultrasound was performed, revealing a well-defined lumbar hernia with a wide hernia neck. The hernia sac contained epiploic fat and digestive content, confirming a communicating hernia (Fig. 2). No signs of strangulation or bowel obstruction were observed.

**Fig. 3:**
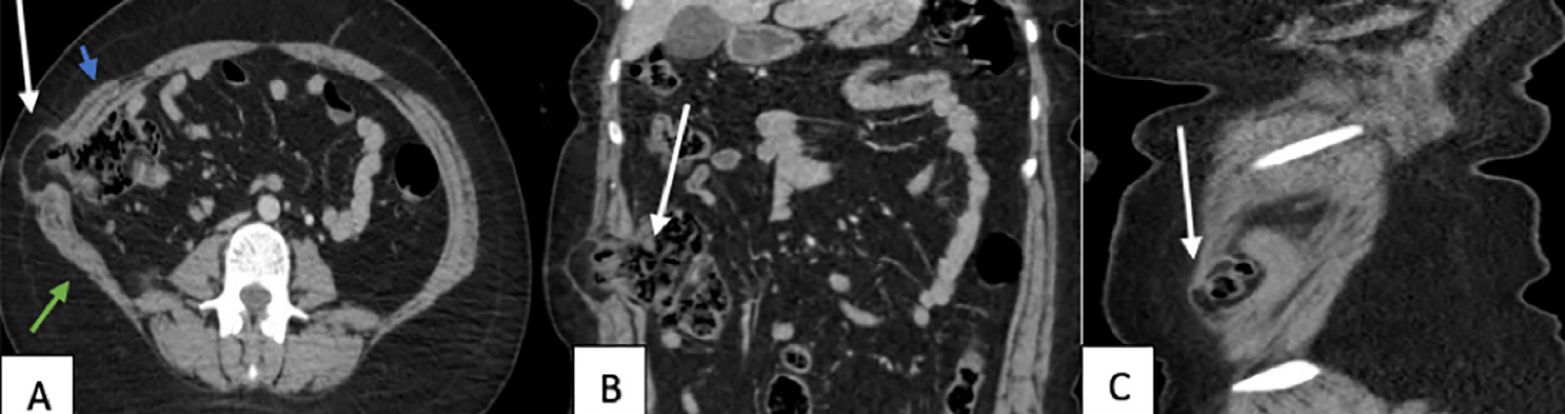
Contrast-enhanced CT scan in the portal phase – axial view (A) with coronal and sagittal reconstructions (B and C). A parietal defect (white arrow) is seen between the external oblique muscle anteriorly (blue arrow) and the latissimus dorsi posteriorly (green arrow) at the level of the inferior lumbar triangle, containing omental and digestive structures without signs of distress.

To further characterize the size, content, and anatomical location of the hernia, a contrast-enhanced CT scan of the abdomen and pelvis was obtained. The CT images confirmed the presence of an inferior lumbar hernia (Jean-Louis Petit hernia) with herniation of epiploic fat and a portion of the digestive tract (Fig. 3). The hernia neck was wide, and there were no complications such as incarceration, strangulation, or obstruction. No additional intra-abdominal pathology was identified.

Given the risk of progressive enlargement, complications such as bowel obstruction or strangulation, and the potential impact on the patient's quality of life, the patient was referred for elective surgical repair. She underwent successful open hernioplasty with mesh reinforcement via a posterior approach. Intraoperative findings confirmed the presence of a wide-necked inferior lumbar hernia containing epiploic fat and a segment of noncompromised bowel. The hernia contents were reduced, and a polypropylene mesh was placed to reinforce the defect.

The postoperative course was uneventful, and the patient was discharged on the third postoperative day with recommendations to avoid strenuous activity for several weeks. At her 1-month follow-up, she reported complete resolution of the lumbar bulge and no recurrence of symptoms. Clinical examination revealed good healing without signs of infection or recurrence. She remained asymptomatic at her 3-month follow-up, with no evidence of recurrence on clinical or sonographic evaluation.

## Discussion

Lumbar hernias are rare conditions. Since the first report by Garangeot RJC in 1731, only around 300 cases have been documented. It has been recognized since 1920 that inferior lumbar hernias (also known as hernias of Jean-Louis Petit, named after the French anatomist who described the lower lumbar surgical anatomy in 1783) are the most common type [3].

The Jean-Louis Petit hernia is characterized by the protrusion of intraperitoneal or extraperitoneal contents into the inferior lumbar triangle (also known as the Jean-Louis Petit triangle). This triangle is defined by the posterior edge of the external oblique anteriorly, the anterior edge of the latissimus dorsi posteriorly, and the iliac crest inferiorly, which forms the base of the triangle. Superficially, it is bounded by the superficial fascia, while its deep boundaries are formed by the internal oblique, fibers of the transversus abdominis, and the posterior layer of the thoracolumbar fascia [1,4].

Congenital forms are rare (20%) [5], whereas acquired lower lumbar hernias are more frequent. Primary acquired hernias (55%) develop spontaneously without specific triggers, with risk factors including advanced age, extreme weight changes, chronic illness, muscle atrophy, and strenuous activity. Secondary acquired hernias (25%) result from trauma, surgery, infections, or conditions such as retroperitoneal hematomas or fractures of the iliac crest [6]. The typical age of affected patients by the Jean-Louis Petit hernia ranges between 50 and 70 years [7]. Our patient falls within this age range and presented with a primary acquired hernia, without any history of surgery or trauma.

The clinical diagnosis of a lumbar hernia requires significant clinical suspicion. It largely depends on the size of the hernia and its contents, which may include retroperitoneal fat, the kidney, or the colon, and less commonly, the small intestine, omentum, spleen, ovary, or appendix [7]. Occasionally, the presentation may resemble that of a lipoma [8]. A lumbar hernia should be considered when a mass appears that is induced by coughing or straining, typically reducible, and tends to disappear when the patient is in a supine position. In our case, the mass's reducibility was crucial in distinguishing the hernia from a lipoma.

Patients may also report a swelling, which may sometimes be accompanied by pain at the site. Over time, the swelling can increase in size, leading to noticeable asymmetry of the trunk [3]. When the hernia contains intestinal loops, bowel sounds may be audible on auscultation, and strangulation can cause symptoms like nausea, vomiting, abdominal distension, and an irreducible mass. If the content is renal, the patient may present with urinary symptoms such as hematuria, oliguria, or renal colic.

In addition to a lipoma, the differential diagnoses to consider, depending on the clinical context, include an abscess, a hematoma, or a soft tissue tumor [3,9].

The differential diagnosis can also be considered with other types of hernias, which are distinguished based on their location. The most common are inguinal hernias, accounting for approximately 75% of abdominal wall hernias. They are divided into direct inguinal hernias (protruding through the posterior wall of the inguinal canal) and indirect inguinal hernias (passing through the deep inguinal ring). Umbilical hernias result from a defect in the closure of the umbilical ring, while linea alba hernias are located along the midline between the xiphoid process and the umbilicus. Another form, Spigelian hernia, develops at the junction between the semilunar line and the arcuate line.

Grynfeltt’s hernia is located in the superior lumbar triangle, which is bounded by the internal oblique muscle, the lower border of the 12th rib, and the quadratus lumborum muscle. In contrast, Jean-Louis Petit's hernia, as in our case, occurs in the inferior lumbar triangle.

Several paraclinical investigations can be performed based on the clinical presentation. Imaging is essential in diagnosing and managing lumbar hernias, especially when clinical findings are inconclusive or complications like strangulation are suspected.

From a radiologist’s perspective, the goal is to identify and characterize the hernia sac, its contents, and the exact location and size of the fascial defect, while also differentiating it from other abdominal wall or retroperitoneal pathologies. For instance, lateral or oblique radiographs of the lumbar region may be obtained, which can reveal gas-filled bowel loops located outside the abdominal cavity if the hernia contains intestinal content. However, these findings are nonspecific and may be subtle or absent in soft-tissue-containing hernias.

Ultrasound can be used as an initial imaging modality, especially for detecting small, reducible hernias or differentiating them from other soft tissue masses like lipomas. It is a dynamic tool that enables the real-time assessment of hernia content and its reducibility, particularly with maneuvers such as coughing or straining. Sonographic features that help differentiate a hernia from a lipoma include the presence of peristalsis, vascularity on Doppler, and changes in shape or position with Valsalva maneuver. However, its limited field of view and operator dependency make it less reliable for complex cases or when deeper anatomical structures are involved [7]. Nevertheless, dynamic ultrasound has proven valuable in rare and atypical presentations. A notable case is the epigastric herniation of the stomach diagnosed by dynamic ultrasound, highlighting the importance of this modality in evaluating unusual hernias [10].

Computed tomography (CT) is considered the gold standard for the evaluation of lumbar hernias. It provides detailed anatomical visualization, allowing precise identification of the hernia's size, location, and content, which may include retroperitoneal fat, kidney, colon, or other less common structures like the small intestine, spleen, or omentum. CT typically reveals a discontinuity in the posterolateral abdominal wall musculature—frequently between the external oblique, internal oblique, and latissimus dorsi in Petit hernias—accompanied by herniation of abdominal or retroperitoneal contents. CT is also invaluable for assessing associated complications, such as bowel obstruction or strangulation [2]. In our case, the CT scan confirmed the diagnosis of a Jean Louis Petit hernia and provided a detailed characterization of the defect.

Magnetic resonance imaging (MRI) may be employed in selected cases, particularly when soft tissue differentiation is required or when evaluating complications involving adjacent structures. It is especially useful for identifying hernias that may mimic tumors or other masses, such as abscesses or hematomas. MRI may show herniated fat or viscera with high soft-tissue contrast, and helps distinguish hernias from neoplastic lesions through multiplanar evaluation and tissue characterization.

In imaging, the other rare forms of abdominal wall hernias, such as Spigelian, obturator, and interparietal hernias, require particular attention. These hernias can be challenging to diagnose, with Spigelian hernias occurring along the semilunar line, obturator hernias presenting with medial thigh or groin pain, and interparietal hernias involving herniation between the layers of the abdominal wall. CT and MRI are crucial for identifying their precise location and contents, allowing differentiation from other abdominal or retroperitoneal pathologies.

Accurate imaging is also critical for preoperative planning, guiding the surgical approach and determining whether laparoscopic or open repair is most appropriate. Based on the imaging results, the surgical strategy is adjusted to meet the patient’s specific needs.

Surgery aims to repair the defect and reconstruct an abdominal wall that is both flexible and resistant to daily physical stress, especially in cases of intestinal content due to the risk of strangulation [11]. Techniques include anatomical closure, reinforcement with musculofascial flaps, and mesh placement via retroperitoneal or transabdominal laparoscopic approaches. Laparoscopy offers benefits such as shorter hospital stays and fewer postoperative complications but carries a higher risk of intraoperative complications [1]. Our patient was referred for elective surgical repair to reduce the risk of future complications.

## Conclusion

Although rare, lumbar hernias pose significant clinical challenges due to their variable symptoms and potential complications. Imaging plays a vital role in their management, with CT scans being the gold standard for diagnosing and planning treatment. CT provides detailed insights into the hernia's size, contents, and any associated complications, facilitating accurate treatment planning and guiding appropriate surgical intervention.

## Patient consent

Written informed consent was obtained from a legally authorized representative(s) for anonymized patient information to be published in this article.
